# Progress in Stereoselective Construction of C–C Bonds Enabled by Aldolases and Hydroxynitrile Lyases

**DOI:** 10.3389/fbioe.2021.653682

**Published:** 2021-04-21

**Authors:** Mi Liu, Dan Wei, Zexing Wen, Jian-bo Wang

**Affiliations:** ^1^Key Laboratory of Chemical Biology and Traditional Chinese Medicine Research (Ministry of Education), College of Chemistry and Chemical Engineering, Hunan Normal University, Changsha, China; ^2^Key Laboratory of Phytochemistry R&D of Hunan Province, College of Chemistry and Chemical Engineering, Hunan Normal University, Changsha, China

**Keywords:** biocatalysis, enantioselectivity, hydroxynitrile lyases, aldolases, C-C bond formation

## Abstract

The creation of C–C bonds is an effective strategy for constructing complex compounds from simple synthetic blocks. Although many methods have been developed for C–C bond construction, the stereoselective creation of new C–C bonds remains a challenge. The selectivities (enantioselectivity, regioselectivity, and chemoselectivity) of biocatalysts are higher than those of chemical catalysts, therefore biocatalysts are excellent candidates for use in stereoselective C–C bond formation. Here, we summarize progress made in the past 10 years in stereoselective C–C bond formation enabled by two classic types of enzyme, aldolases and hydroxynitrile lyases. The information in this review will enable the development of new routes to the stereoselective construction of C–C bonds.

## Introduction

Stereoselective construction of C–C bonds enables the formation of complex, enantiomerically pure, multifunctional macromolecular compounds from simple synthetic blocks. This provides an effective pathway for the production of important compounds such as natural products, pesticides, and pharmaceutical intermediates. The conciseness, environmental friendliness, and atom economy of synthetic methods have become important aspects of synthetic chemistry. Chemists have developed many excellent methods for constructing C–C bonds, such as Heck coupling (Andrus et al., 2002), Suzuki coupling (Suzuki, 2004), and Stille coupling (Jin and Lee, 2010). These methods can achieve high conversions and selectivities, but they involve toxic organic halogenations, require harsh reaction conditions, and have low atom economies. Enzymatic methods generally require milder conditions and have better atom economies than chemical methods (Seoane, 2000). Various types of enzyme that catalyze the enantioselective construction of C–C bonds have been reported (Clapés, 2016), such as methyltransferase (Mahanta et al., 2017; Sato et al., 2017), aldolases (Wagner et al., 1995), diels-alderases (Pohnert, 2001), hydroxynitrile lyases (HNLs) (Effenberger et al., 2000), and transketolases (Turner, 2000). A wide range of enzymes have been investigated but because of space limitations, we have concentrated on aldolases and HNLs.

Aldolases catalyze the selective condensation of aldols to produce C–C bonds, usually with the simultaneous creation of two new chiral centers (Davis and Boyer, 2001; Windle et al., 2014). HNLs are aldehyde lyases and are classified as C–C lyases. They mainly originate from plants and occur in bacteria and arthropods. They catalyze C–C bond formation via addition of hydrogen cyanide with an aldehyde/ketone, or nitroalkanes with aldehydes, to form cyanohydrins or nitro alcohols (Schmidt and Griengl, 1999). In this review, we summarize research progress in C–C bond formation enabled by aldolases and HNLs in the last 10 years and focus on their stereoselective synthetic applications.

## Aldolases

**FIGURE 1:**
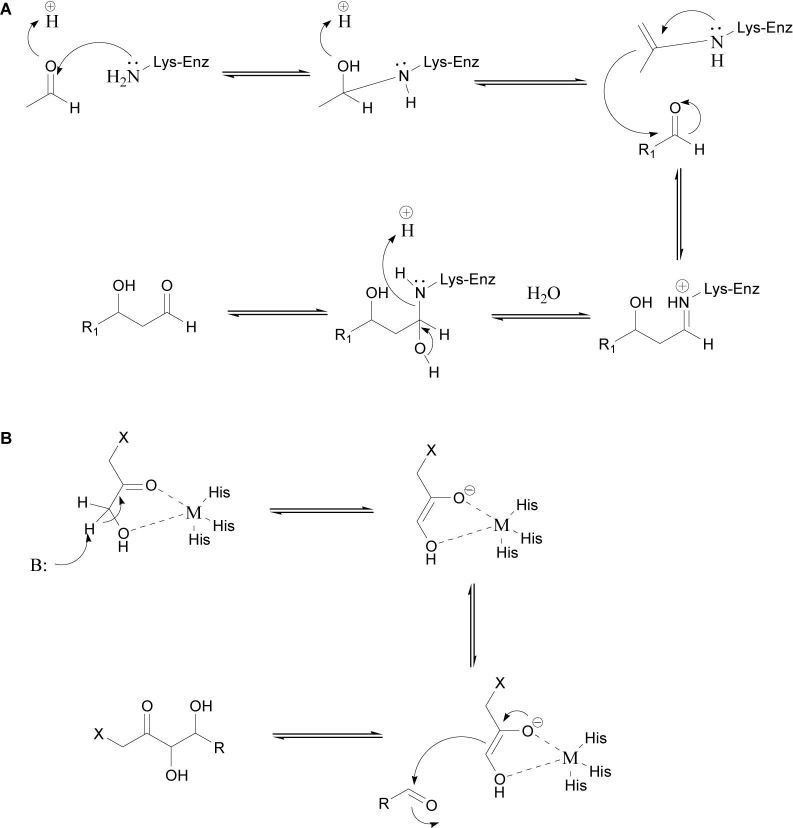
General reaction mechanisms of class I **(A)** (Haridas et al., 2018) and class II **(B)** aldolases (Falcicchio et al., 2014).

The use of aldolases has enabled significant developments in enantioselective C–C bond formation (Clapes and Garrabou, 2011). The aldol condensation reaction, which can be catalyzed by an aldolase, involves the addition of a nucleophilic donor (e.g., an aldehyde or ketone) to an electrophilic acceptor (usually an aldehyde). Aldolases can generally tolerate a broad range of acceptor substrates but have high specificity for the donor substrate (Güclü et al., 2016). Aldolases are usually divided into four categories based on the donor specificity: acetaldehyde-dependent, pyruvate/2-oxobutyrate-dependent, glycine-dependent, and dihydroxyacetone phosphate-dependent aldolases. On the basis of their catalytic mechanisms, aldolases can be divided into two categories, namely class I and class II aldolases. Class I aldolases do not require cofactors and use a conserved lysine as the active site. The amino group of this lysine forms a Schiff base intermediate with the active carbonyl group on the donor. The Schiff base intermediate is converted into an enamine nucleophile, which then attacks the carbonyl carbon on the acceptor to form a C–C bond (Figure 1A). Class II aldolases require divalent metal ion cofactors such as Zn^2+^, Fe^2+^, Mg^2+^, or Co^2+^, which act as Lewis acids and activate nucleophiles by coordinating to the carbonyl group of the donor (Figure 1B) (Clapés et al., 2010; Marsden et al., 2019).

The use of aldolases gives three types of product, monoalcohols, polyalcohols, and amino alcohols. Chiral alcohols are important building blocks in the production of chiral drugs, agrochemicals, and fine chemicals (Iwasaki et al., 1989; Ma et al., 2010; Yang et al., 2020). Polyalcohols are more complex than monoalcohols and more difficult to synthesize via chemical methods. Amino alcohols contain a β-hydroxy-α-amino acid structural unit. They are used as precursors in the production of pharmaceuticals and agrochemicals (Steinreiber et al., 2007), such as chloramphenicol (Ehrlich et al., 1947), vancomycin (Yim et al., 2014), thiamphenicol, and florfenicol (Steinreiber et al., 2007). Here, we will discuss aldolases on the basis of their products, in the order monoalcohols, polyalcohols, and amino alcohols.

**SCHEME 1:**
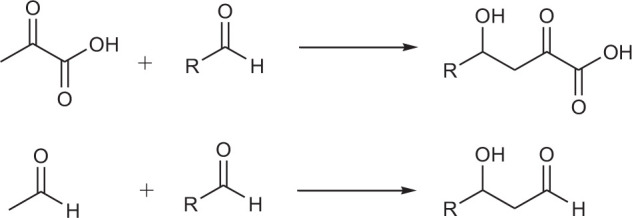
Examples of monoalcohol production.

Monohydric alcohols are usually produced by using aldehydes (ketones) as nucleophiles to attack aldehydes (ketones). This generates monohydric alcohol products (Scheme 1).

Howard et al. (2016) reported that *trans-o*-hydroxybenzylidene pyruvate hydratase-aldolase catalyzes the condensation reaction of sodium 3-fluoropropionate with an aromatic aldehyde to form valuable fluorine-containing monobasic chiral alcohols. Esters were subsequently synthesized by a chemical method (Howard et al., 2016). Highly enantioselective aromatic products were obtained via this chemo-enzymatic method (Table 1, entries 1–12). Fang et al. used five different enzymes from the type II HpcH aldolase family (EcGarL, EcRhmA, and EcHpcH from *Escherichia coli*, and SwHpcH1 and SwHpcH2 from *Sphingomonas wittichi*) to expand the condensation reaction between fatty aldehydes and 3-fluoropropionic acid (Fang et al., 2019). Among the reactive receptor substrates (Table 1, entries 13–18), EcGarL catalyzed the production of 2-fluoro-3-hydroxysuccinate with high reactivity (99%) and enantioselectivity (99%) (Table 1, entry 14).

**TABLE 1:**
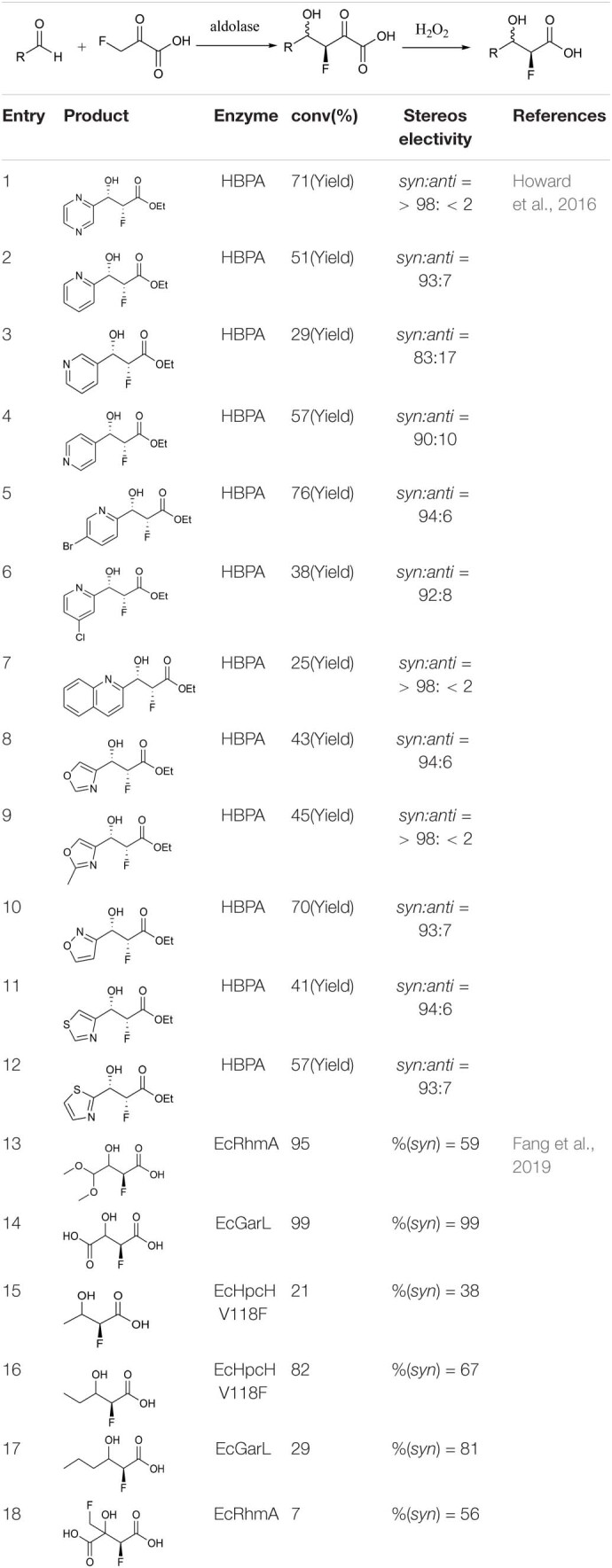
Reaction data for some monoalcohol products.

**SCHEME 2:**
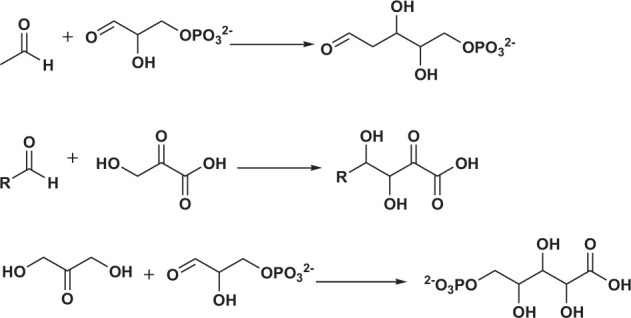
Examples of polyol production.

Polyol products are generally obtained via the reaction of aldehydes (ketones) or alcohol-containing aldehydes (ketones) as nucleophiles with one or more alcohol-containing electrophiles (Scheme 2).

Polyols are the most common products in aldolase-catalyzed reactions. These reactions mainly produce aliphatic-chain polyol products. Yang et al. (2017) engineered D-fructose-6-phosphate aldolase (FSAA) from *E. coli* to increase the reactivities of four aromatic aldehyde receptor substrates with hydroxyacetone. The FSAA Q59T mutant showed the best catalytic efficiency (Supplementary Table 1, entries 1–4) and gave the highest conversion rate, 93%, in the condensation of picolinaldehyde and hydroxyacetone (Supplementary Table 1, entry 4).

It has been reported that wild-type FSAA can catalyze the production of fatty-chain polyhydroxylated compounds, and the mutant A196S can increase the enzymatic activity by stabilizing the Schiff base intermediate. Castillo et al. (2010) used the A196S mutant to investigate the reactions of donor substrates such as dihydroxyacetone, hydroxyacetone, and glycolaldehyde with some acceptor substrates (Supplementary Table 1, entries 5–7, 13–16, and 22). Although aldolases can generally accept a broad range of receptors, the scope of the FSA A196S mutant is still not extensive. For both dihydroxyacetone and hydroxyacetone donor substrates, Gutierrez et al. (2011) redesigned the mutation to obtain the mutant FSA A129S/A165G, which expanded the range of receptor substrates to α-substituted aminoaldehydes (Supplementary Table 1, entries 8–12, and 17–21). Subsequently, glycolaldehyde was used as a nucleophile in the design of FSA mutations and it was discovered that the mutations at positions L107 and A129 are crucial for its donor activity and selectivity. The A165G mutation enhances the reactivity toward aldehydes of low reactivity (Szekrenyi et al., 2014). Szekrenyi et al. (2015) successfully synthesized aldoses with three to six carbons by gradually adding glycolaldehyde to a series of electrophilic substrates in one pot. On the basis of existing mutants, saturation mutation at position S166 was performed to obtain the highest reactivity and stereoselectivity (Supplementary Table 1, entries 23–34) (Szekrenyi et al., 2015). For example, FSA A129T/A165G catalyzed the formation of *L*-xylose with strict stereoselectivity and 98% conversion (Supplementary Table 1, entry 24). In addition to modifying the acceptor substrate scope, much work has been done to expand the donor substrate scope. Güclü et al. (2016) designed FSA mutations and obtained the effective mutants L107A/L163A, which greatly expanded the affinity substrate scope of the enzyme (Supplementary Table 1, entries 35–47) (Güclü et al., 2016). However, until 2017, only substrates with hydroxymethyl moieties were used as the nucleophiles. The breakthrough was achieved by Roldán et al. (2017). They developed four aliphatic ketones (acetaldehyde, acetone, methyl ethyl ketone, and cyclopentanone) as nucleophilic substrates by using the FSA D6H variant; high yields and high stereoselectivities were achieved (Supplementary Table 1, entries 48–51) (Roldán et al., 2017).

Enzymes other than FSA can also generate polyols. 2-Deoxyribose 5-phosphate aldolase (DERA) is an acetaldehyde-dependent enzyme (Haridas et al., 2018). It can accept three affinity substrates (propionaldehyde, acetone, and fluoropropane) as well as the natural nucleophilic substrate acetaldehyde, but the activities are lower than that of acetaldehyde (Barbas et al., 1990; Chen et al., 1992; Wong et al., 1995). Chambre et al. (2019) explored six different nucleophilic substrates (propanol, propionaldehyde, cyclobutanone, cyclopentanone, dihydroxyacetone, and glycolaldehyde) with DERA from *Arthrobacter chlorophenolicus*. A certain degree of complementarity with FSA was observed (Supplementary Table 1, entries 52–57) (Chambre et al., 2019).

Besides the regular donor substrates, nucleophilic substrates containing phosphate groups can also be accepted by aldolases. For dihydroxyacetone phosphate-dependent aldolases, dihydroxyacetone with a phosphate group is used as the nucleophilic substrate. Rhamnose-1-phosphate aldolase (RhuAs) is a dihydroxyacetone phosphate-dependent aldolase that can catalyze the cross-aldehyde condensation between dihydroxyacetone phosphate and dihydroxyacetone. Laurent et al. (2018) explored the scope of electrophilic substrates for this type of enzyme, and confirmed that it has strong substrate promiscuity (Supplementary Table 1, entries 58–67).

Fang et al. (2019) used five different pyruvate aldolases to catalyze the introduction of fluorine atoms to form monoalcohol products. The same method was used to form valuable fluorine-containing polyhydroxylated products (Supplementary Table 1, entries 68–72) (Fang et al., 2019).

**SCHEME 3:**
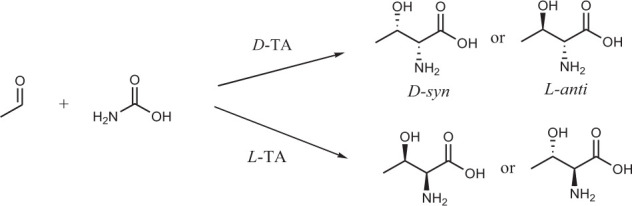
Isomeric products of threonine aldolase-catalyzed condensation reaction of acetaldehyde and glycine.

The reactions that produce amino alcohols involve attacking aldehydes with amino acids as nucleophiles. The classic aldolase that catalyzes this reaction is threonine aldolase (TA). TA is a pyridoxal-5-phosphate-dependent enzyme that reversibly catalyzes aldol reactions between glycine and aldehyde to create two new stereocenters (Scheme 3).

Most TAs prefer aromatic aldehydes as acceptors, and the donor is generally glycine. Baer et al. (2011) investigated the substrate tolerances of two TAs, from *E. coli* and *Saccharomyces cerevisiae*, by increasing the aldehyde concentration to 250 mM. Even when the substrate concentration was increased to 250 mM, the *L*-TA from *E. coli* can still catalyze the stereoselective addition of mono-substituted aromatic aldehydes and glycine with good reactivity and stereoselectivity (Table 2, entries 2, 4, 8, and 9). In particular, the reaction of *o*-chlorobenzaldehyde with glycine gave a good conversion rate, >95%, and a fair diastereoselectivity (dr; *syn*/*anti* = 80:20; Table 2, entry 2). Blesl et al. (2018) explored the substrate scope of TA from *Pseudomonas* spp. A series of aromatic aldehydes with electron-withdrawing substituents (e.g., halo, nitro) and an electron-donating substituent (methoxy), and a few aliphatic aldehydes were tested (Table 2, entries 1–6, 16, and 22–30) (Blesl et al., 2018). Most of the substrates were reactive, but the conversion rates were relatively low, mostly below 50%. In 2020, Wang et al. explored the substrate profile of *L*-TA from *Actinocorallia herbida* (AhLTA). The conversion rates for AhLTA were significantly higher than those for a series of receptor substrates explored by Blesl et al. (2018), and most of them exceeded 50% (Table 2, entries 1–5, and 16) (Wang et al., 2020). The conversion rate for the AhLTA- catalyzed reaction of *o*-nitrobenzaldehyde and glycine was 81%.

**TABLE 2:**
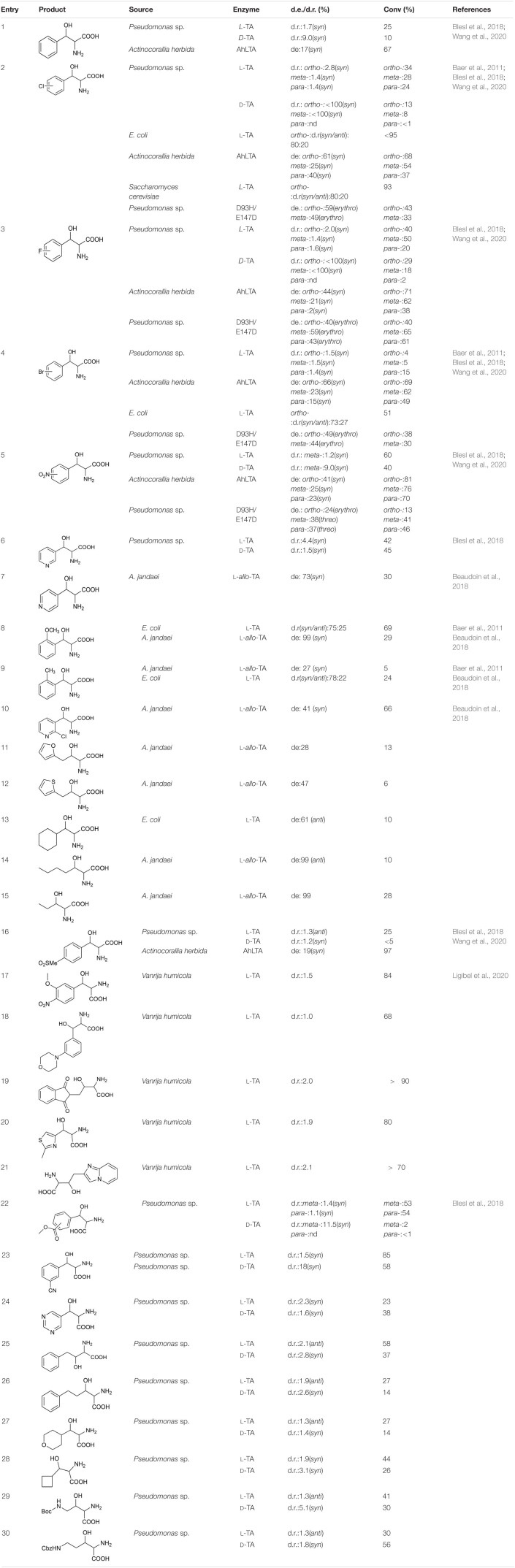
Receptor substrate and glycine reaction data.

The substrates discussed above are basically small aromatic aldehydes. There have been few reports of aldolase-catalyzed reactions of large aromatic aldehydes. Ligibel et al. (2020) extracted TA from *Vanrija humicola* ATCC 20265 (*L*-TA_1), used it to catalyze the reactions of a series of large aromatic aldehydes, and separated the corresponding products. The conversion rates of most of the substrates were high (Table 2, entries 17–21). For example, when *N*-(2-acetaldehyde)phthalimide was used as the substrate, a <90% conversion rate was achieved (Table 2, entry 19).

In addition to accepting aromatic aldehydes, TA can also accept aliphatic aldehyde and heterocyclic aldehyde substrates in these catalytic reactions. Beaudoin et al. (2018) successfully reacted some fatty aldehyde and heterocyclic aldehyde receptor substrates with glycine. Three enzymes (derived from *A. jandaei*, *E. coli*, and *T. maritima*) were selected. TA from *A. jandaei* gave the best results. Although the stereoselectivity was good, the conversion rate was low (Table 2, entries 7–15).

Although TA can perfectly control the C_α_ stereoselectivity, it is difficult to improve the C_β_ stereoselectivity, and there have been few reports of such improvements. In the most recent study of C_β_-stereoselectivity modulation, Chen et al. (2019) performed protein engineering on *L*-TA from *Pseudomonas* spp. and obtained a mutant, D93N/E147D, which catalyzed the production of C_β_-stereoselective reversed products (Table 2, entries 2–6).

There is a wide range of TA receptors, but the donor is generally glycine and the donor substrate scope is narrow. Earlier studies showed that in addition to glycine, TA can tolerate *D*-alanine, *D*-serine, and *D*-cysteine as donor substrates (Table 3, entries 1–12) (Fesko et al., 2010). Blesl et al. (2018) tried to expand the donor substrate scope for TAs from *Pseudomonas* spp. However, in addition to the four donor substrates previously reported, only *L*-2-aminobutyric acid and 3,3,3-trifluoro-*L*-alanine showed weak activity (Table 3, entries 13–16).

**TABLE 3:**
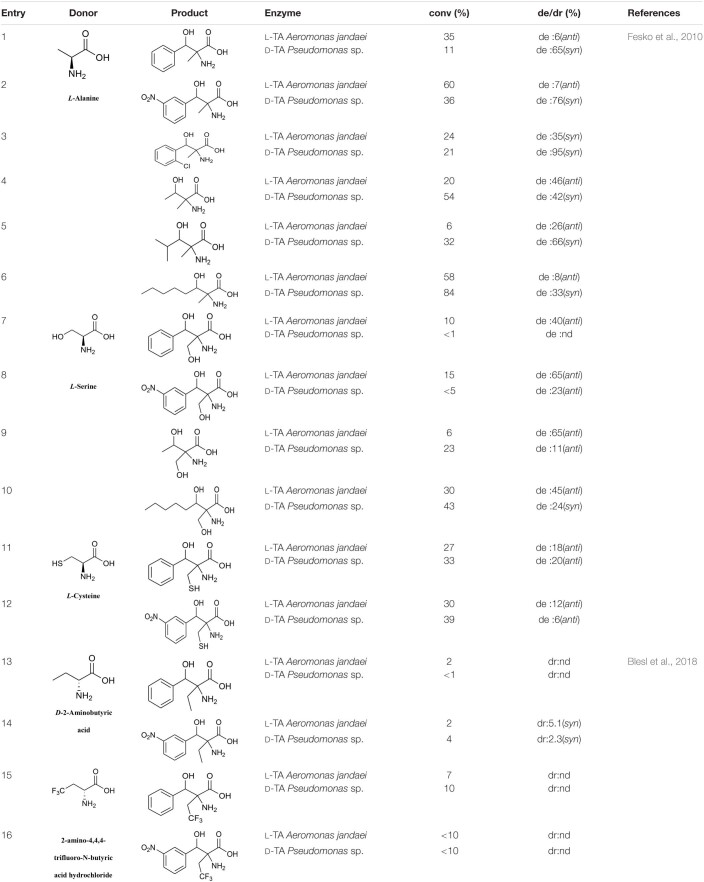
Threonine aldolase-catalyzed reactions of various donor substrates.

## Hydroxynitrile Lyases

**FIGURE 2:**
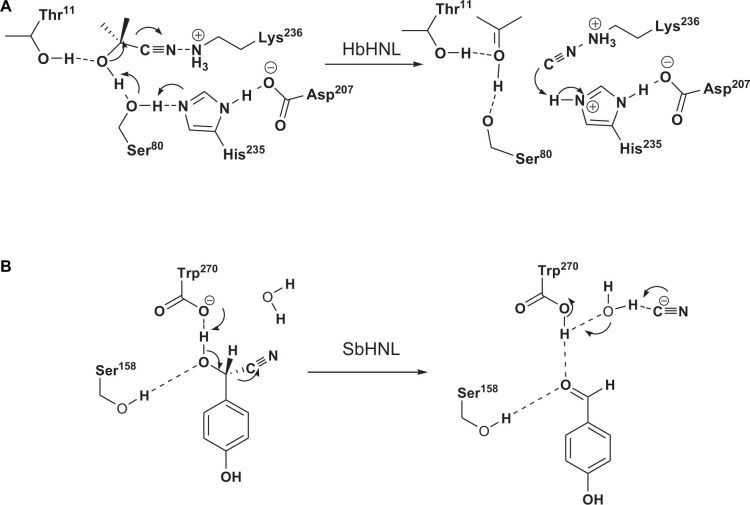
Proposed reaction mechanisms for α/β-hydrolase HNL (HbHNL) **(A)** and carboxypeptidase HNL (SbHNL) **(B)**.

Hydroxynitrile lyases crystal structures are similar to those of proteins in seven superfamilies: glucose-methanol-choline oxidoreductase (Dreveny et al., 2001), α/β-hydrolases (Hickel et al., 1996), carboxypeptidases (Lauble et al., 2002), zinc-dependent HNL (Trummler and Wajant, 1997), bacterial cupin (Hajnal et al., 2013), bet v1 folding HNL (Lanfranchi et al., 2017), and dimeric α + β barrel folds (Motojima et al., 2018). The catalytic mechanism involves general acid/base catalysis, but the details for each enzyme are different (Fesko and Gruber-Khadjawi, 2013). For example, HbHNL, which is an α/β-hydrolase, catalyzes the target reaction via a triad catalytic core (Figure 2A). SbHNL is a carboxypeptidase; its catalytic mechanism involves use of the carboxyl group of C-terminal Trp270 as a catalytic base for extracting protons from cyanohydrin (Figure 2B) (Gruber and Kratky, 2004).

**FIGURE 3:**
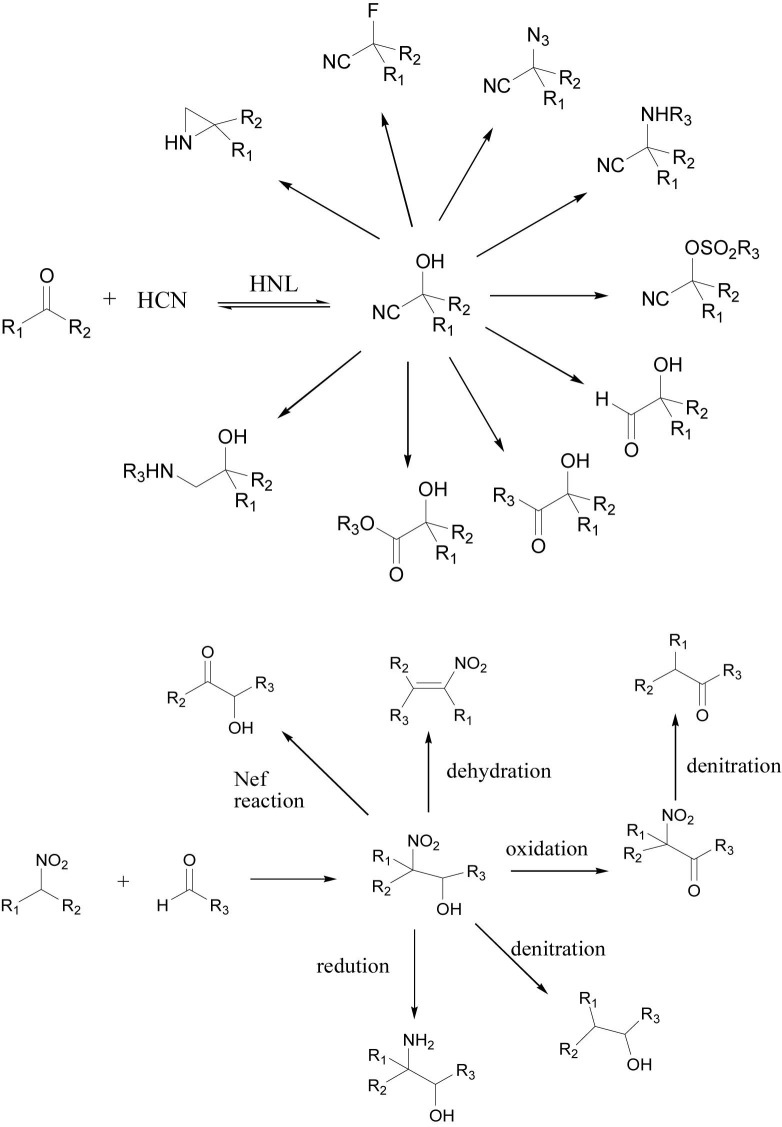
Derivation of cyanohydrin and nitro alcohol.

Hydroxynitrile lyases are widely used in the industrial production of enantiopure cyanohydrins, which are well-known drug precursors. In addition to forming cyanohydrins, HNLs can catalyze the enantioselective addition of nitroalkanes and aldehydes to generate nitro alcohols (Yuryev et al., 2010). Both cyanohydrins and nitro alcohols can be used to construct useful compounds (Gaggero, 2019). For example, nitro alcohols can be converted to 1,2-amino alcohols, conjugated nitroalkenes, α-hydroxy carbonyl compounds, and nitro carbonyl compounds (Figure 3) (Milner et al., 2012).

Hydroxynitrile lyases have a limited substrate scope, namely aromatic aldehydes, fatty aldehydes, and fatty ketones, and most HNLs prefer aromatic aldehyde substrates. In 2010, Asano discovered a new type of HNL, PeHNL (from *Passiflora edulis*), which is not highly reactive with benzaldehyde and substituted benzaldehydes (Table 4, entries 1 and 2), but has high reusability. In the fourth cycle, the conversion ratio and enantiomeric excess were still 26.4 and 98.7%, respectively, when benzaldehyde was used as the substrate (Ueatrongchit et al., 2010). In 2014, Steiner’s team obtained *R*-GtHNL-M (A40H/V42T/Q110H, an HNL derived from *Granulicella tundricola*) by protein engineering. It gave good enantioselectivity and activity in reactions with various aromatic aldehydes (Table 4, entries 1, and 3–7) (Wiedner et al., 2015). The same team discovered an HNL (AcHNL) in *Acidobacterium capsulatum*. Its activity and enantioselectivity in reactions with benzaldehyde were only slightly inferior to those of the GtHNL mutant, and it showed good enantioselectivity with large volumes of 3-phenoxybenzaldehyde (Table 4, entries 1 and 7) (Wiedner et al., 2014). Dadashipour et al. (2015) found that another HNL, ChuaHNL from *Chamberlinius hualienensis*, showed broad activity toward a series of aromatic aldehydes with electron-withdrawing and electron-donating substituents (Table 4, entries 8–12).

**TABLE 4:**
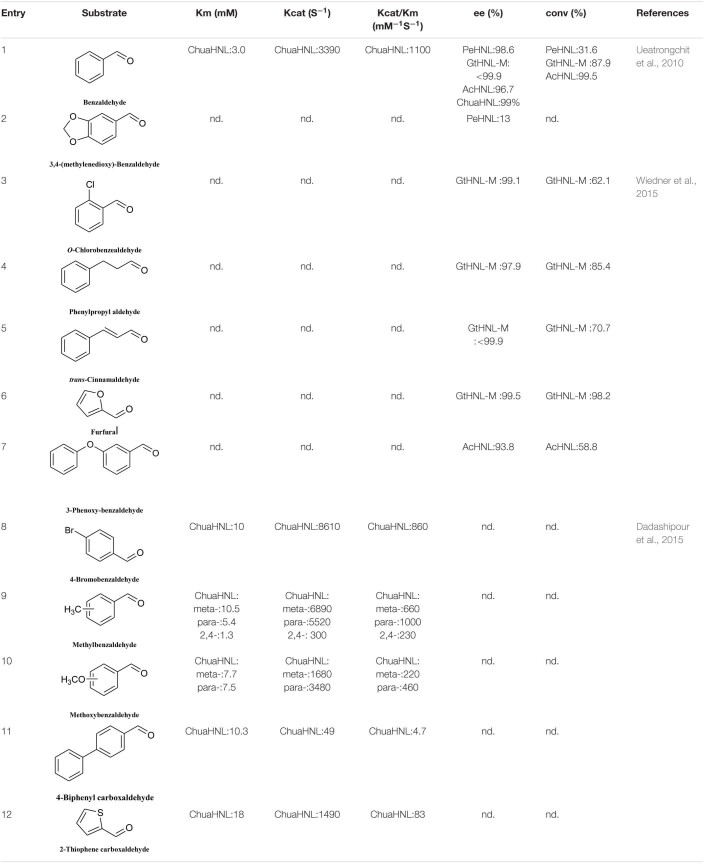
Substrate scope of *R*-HNLs.

*nd, not described.*

The number of reported *S*-HNLs is much lower than that of *R*-HNLs, and most of them have drawbacks such as a narrow substrate scopes and low activities. Dadashipour et al. (2011) isolated a new type of *S*-HNL from *Bamarispermum montanum* (BmHNL) plants. BmHNL has a broad substrate scope and can accept aromatic aldehydes with electron-withdrawing and electron-donating groups on the aromatic rings (Table 5, entries 1–8) (Dadashipour et al., 2011). Although the substrate scope is broad, the reactivity and the enantioselectivity are low. In 2016, a mutant, H103C/N156G, was created by protein engineering. In tests with benzaldehyde as the substrate, the ee value increased to 93% from the wild-type value of 55% (Asano and Kawahara, 2016). Jangir et al. (2018) used BmHNL lysate in a two-phase system to synthesize cyanohydrin and tested many aromatic compounds. The reported activities with some substrates were lower than those reported for *S*-HNL, but this enzyme shows strong substrate tolerance (Table 5, entries 10–15).

**TABLE 5:**
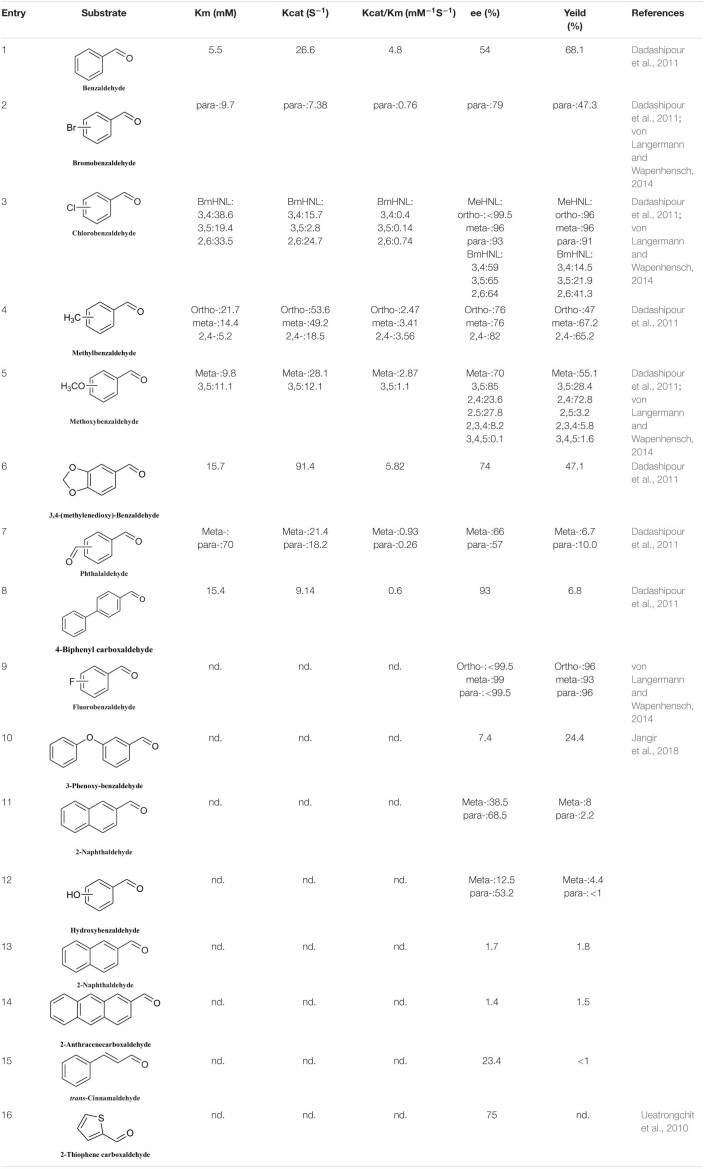
Substrate scope of *S*-HNLs.

*nd, not described.*

Another *S*-selective HNL, MeHNL, was isolated from *Manihot esculenta*. von Langermann and Wapenhensch (2014) used this enzyme in a biocatalytically active static emulsion technique to catalyze a series of reactions of substituted aromatic aldehydes to the corresponding cyanohydrins. Some of the ee values and conversion rates for halogen-substituted aromatic aldehydes were greater than 90% (Table 5, entries 2, 3, 5, and 9). These *S*-HNL enzymes produce *S*-products. In some cases, *R*-HNLs can also catalyze the production of *S*-products from specific substrates. PeHNL usually produces *R*-cyanohydrin, but when a sulfur-heterocyclic aldehyde was used as the substrate, *S*-cyanohydrin was produced, with an ee value of 75% (Table 5, entry 16) (Ueatrongchit et al., 2010).

The HNL tolerance for aliphatic aldehydes and ketones is much poorer than that for aromatic aldehydes. PeHNL can catalyze the conversion of short-chain trace aldehydes and cyclohexylformaldehyde to the corresponding *R*-cyanohydrins, but the conversion ratios and enantioselectivities are low (Table 6, entries 1–4) (Ueatrongchit et al., 2010). *S*-BmHNL tolerates a broad range of aromatic aldehydes and can accept partial long-chain fatty aldehydes. However, in terms of ketone substrates (Table 6, entries 5–7), it can only catalyze the conversion of 5-methyl-2-hexanone to the corresponding *S*-cyanohydrin (Table 6, entry 8) (Dadashipour et al., 2011), and the reactivity and enantioselectivity are low. In the case of MeHNL, when long-chain (C_5_–C_8_) aliphatic ketones are used, the enantioselectivity is good, but the conversion rate gradually decreases with increasing carbon chain length (Table 6, entries 9–12) (von Langermann and Wapenhensch, 2014).

**TABLE 6:**
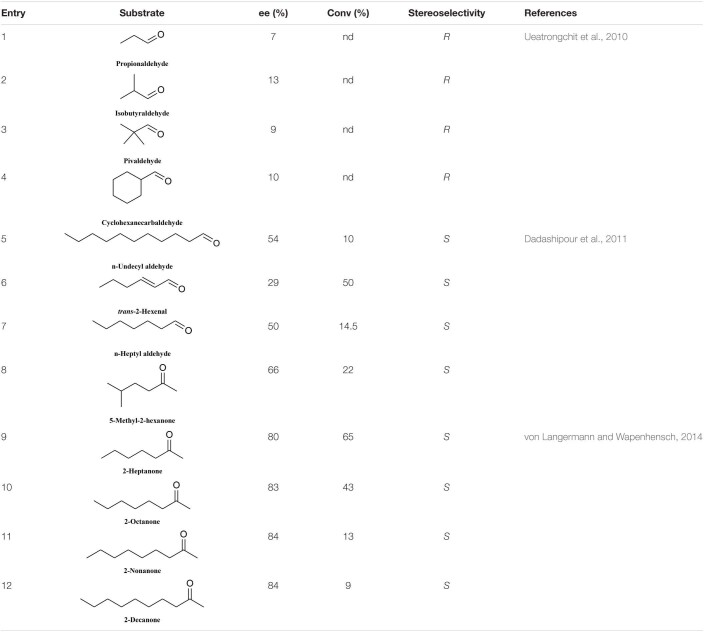
Conversion rate and enantiomeric excess data for some fatty aldehydes and ketones.

*nd, not described.*

In addition to catalyzing reactions between hydrogen cyanide and aldehydes/ketones, HNLs can create new C–C bonds by catalyzing the addition reaction between aldehydes and nitroalkanes. Bekerle-Bogner et al. (2016) used AcHNL and GtHNL mutants to catalyze the stereoselective addition of benzaldehyde or halogenated benzaldehydes and nitromethane. Both of these enzymes and their mutants can catalyze the production of *R*-nitro alcohols. The reactions of benzaldehyde and chlorobenzaldehyde, respectively, with nitromethane gave relatively high conversion ratios and ee values (Table 7, entries 1 and 2). Two fatty aldehydes were tested, and their reactivities and enantioselectivities were both higher than those of aromatic aldehydes (Table 7, entries 3 and 4) (Bekerle-Bogner et al., 2016).

**TABLE 7:**
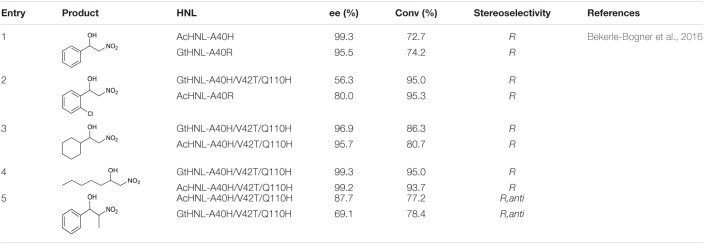
Some nitro alcohol products.

In addition to reacting with nitroalkanes to produce nitrates containing one chiral center, HNLs can also react with nitroethane to produce two chiral centers. The enantioselectivity of the HNL-catalyzed production of two chiral centers was lower than that of the production of a single chiral center (Table 7, entry 5) (Bekerle-Bogner et al., 2016).

## Conclusion

The formation of C–C bonds is important in chemistry, but traditional chemical methods are usually not environmentally friendly. Enzymatic methods, which have greater efficiency and are greener, provide a complementary approach to the creation of new C–C bonds. Here, we summarized progress in aldolase-and HNL-catalyzed C–C bond formations in the past 10 years. Although significant progress has been achieved, these enzymes still have some disadvantages such as instability, low activity, and narrow substrate scopes. Industrial applications are therefore still out of reach. We hope that this review can provide enough information to promote the development of this field in the future.

## Author Contributions

J-BW conceived and provided advices for this review, revised and supplemented the whole manuscript. ML conceived and wrote the manuscript. DW and ZW provided the materials for the manuscript writing. All the authors read, approved, and modified the final manuscript.

## Conflict of Interest

The authors declare that the research was conducted in the absence of any commercial or financial relationships that could be construed as a potential conflict of interest.
