# Supplementary material for: Progress in Stereoselective Construction of C–C Bonds Enabled by Aldolases and Hydroxynitrile Lyases
Source: Front Bioeng Biotechnol. 2021 Apr 21;9:653682. doi: 10.3389/fbioe.2021.653682 (PMC8097096; doi:10.3389/fbioe.2021.653682)
Supplement: Supplementary file 1 [file Table_1.docx]

**Supplementary Table. Partially polyhydroxylated products.**

| **entry** | Donor | Acceptor | **product** | **enzyme** | **Conv**  **(%)** | **Stereoselectivity** | **Ref.** |
| --- | --- | --- | --- | --- | --- | --- | --- |
| **1** |   **Hydroxyacetone** |  **Cinnamaldehyde** |  | **FSAA Q59T** | **30** | **-** | [**Yang et al., 2017**](#_ENREF_63) |
| **2** |  | **Bromocinnamaldehyde** |  | **FSAA Q59T** | **5** | **-** |  |
| **3** |  |   **(*E*)-3-(4-nitrophenyl)**  **acrylaldehye** |  | **FSAA Q59T** | **21** | **-** |  |
| **4** |  |   **2-Oxyethyl pyridine** |  | **FSAA Q59T** | **93** | **-** |  |
| **5** |  |   **4-Nitrobutanal** |  | **FSA A196S** | **55** | **-** | [**Castillo et al., 2010**](#_ENREF_8) |
| **6** |  |   **Glycolaldehyde** |  | **FSA A196S** | **70** | **-** |  |
| **7** |  |   **(*S*)-Glyceric acid** |  | **FSA A196S** | **86** | **-** |  |
| **8** |  | ****  **(*S*)-benzyl-1-oxopropanyl-2-**  **carbamate** |  | **FSA A129S/A165G** | **92** | **-** | [**Gutierrez et al., 2011**](#_ENREF_27) |
| **9** |  |   **(*R*)-benzyl-1-oxopropanyl-2-**  **carbamate** |  | **FSA A129S/A165G** | **95** | **-** |  |
| **10** |  |   **Benzyl (2-oxoethyl)**  **carbamate** |  | **FSA A129S/A165G** | **100** | **-** |  |
| **11** |  |   **2-(benzyloxy)acetaldehyde** |  | **FSA A129S/A165G** | **100** | **-** |  |
| **12** |  |   **3-(benzyloxy)propanal** |  | **FSA A129S/A165G** | **96** | **-** |  |
| **13** |   **1,3-Dihydroxy**  **acetone** |   **4-Nitrobutanal** |  | **FSA A196S** | **73** | **-** | [**Castillo et al., 2010**](#_ENREF_8) |
| **13** |  | **(*R*)-2-hydroxy- 4-nitrobutanal** |  | **FSA A196S** | **52** | **-** |  |
| **14** |  |   **Formaldehyde** |  | **FSA A196S** | **68** | **-** |  |
| **15** |  |   **2-Hydroxyacetaldehyde** |  | **FSA A196S** | **80** | **-** |  |
| **16** |  |   **(*S*)-Glyceric acid** |  | **FSA A196S** | **81** | **-** |  |
| **17** |  | ****  **(*S*)-benzyl-1-oxopropanyl-2-**  **carbamate** |  | **FSA A129S/A165G** | **78** | **-** | [**Gutierrez et al., 2011**](#_ENREF_27) |
| **18** |  |   **(*R*)-Benzyl-1-oxopropanyl-2-**  **carbamate** |  | **FSA A129S/A165G** | **95** | **-** |  |
| **19** |  |   **Benzyl (2-oxoethyl)**  **carbamate** |  | **FSA A129S/A165G** | **100** | **-** |  |
| **20** |  |   **2-(benzyloxy)acetaldehyde** |  | **FSA A129S/A165G** | **100** | **-** |  |
| **21** |  |   **3-(benzyloxy)propanal** |  | **FSA A129S/A165G** | **99** | **-** |  |
| **22** |   **Glycoladehyde** |  |  | **FSA A196S** | **38** | **-** | [**Castillo et al., 2010**](#_ENREF_8) |
| **23** |  |   **Glycolaldehyde** |  | **FSA A196G** | **98** | **-** | [**Szekrenyi et al., 2015**](#_ENREF_51) |
|  |  |  |  | **FSA A129T/A165G** | **85** | **-** |  |
| **24** |  |   **Formaldehyde** |  | **FSA WT** | **99** | **-** |  |
|  |  |  |  | **FSA A129T/A165G** | **98** | **-** |  |
| **25** |  |   **3-Hydroxypropionaldehyde** |  | **FSA L107Y/A129G** | **90** | **-** |  |
| **26** |  | ****  **Propionaldehyde** |  | **FSA A129T/A165G/S166G** | **70** | **-** |  |
| **27** |  | ****  **Methoxyacetaldehyde** |  | **FSA A129T/A165G/S166G** | **79** | **-** |  |
| **28** |  | ****  **2-Benzyloxyacetaldehyde** |  | **FSA A129T/A165G/S166G** | **90** | **-** |  |
| **29** |  | ****  **2-(benzylthio)acetaldehyde** |  | **FSA A129T** | **81** | **-** |  |
| **30** |  | ****  **2-Phenoxyacetaldehyde** |  | **FSA A129T/S166G** | **65** | **-** |  |
| **31** |  | ****  **azido-Acetaldehyde** |  | **FSA A129T/S166G** | **80** | **-** |  |
| **32** |  | **Chloroacetaldehyde** |  | **FSA A129T/S166G** | **67** | **-** |  |
| **33** |  | ****  **Propionaldehyde** |  | **FSA A129T/S166G** | **51** | **-** |  |
| **34** |  | ****  **Phenylpropyl aldehyde** |  | **FSA A129T/S166G** | **58** | **-** |  |
| **35** | ****  **Hydroxyacetone** |   **3-Hydroxypropionaldehyde** |  | **FSA L107A/L163A** | **87** | **-** | [**Güclü et al., 2016**](#_ENREF_26) |
| **36** | ****  **1-Hydroxy-**  **2-butanone** |  |  | **FSA L107A/L163A** | **89** | **-** |  |
| **37** | ****  **1-Hydroxy-**  **2-pentanone** |  |  | **FSA L107A/L163A** | **75** | **-** |  |
| **38** | ****  **1-Hydroxy-**  **2-hexanone** |  |  | **FSA L107A/L163A** | **76** | **-** |  |
| **39** | ****  **1-Hydroxy-**  **2-Heptanone** |  |  | **FSA L107A/L163A** | **50** | **-** |  |
| **40** | ****  **1-Hydroxy-4-**  **methyl-2-Pentanone** |  |  | **FSA L107A/L163A** | **28** | **-** |  |
| **41** | ****  **1-Hydroxy-5-**  **methylhexan-2-one** |  |  | **FSA L107A/L163A** | **25** | **-** |  |
| **42** | ****  **1-Hydroxy-4,4-**  **dimethylpentan-2-one** |  |  | **FSA L107A/L163A** | **25** | **-** |  |
| **43** | ****  **Methyl glycolate** |  |  | **FSA L107A/L163A** | **89** | **-** |  |
| **44** | ****  **Ethyl glycolate** |  |  | **FSA L107A/L163A** | **82** | **-** |  |
| **45** | ****  **Glycolic acid propyl ester** |  |  | **FSA L107A/L163A** | **28** | **-** |  |
| **46** | ****  **Isopropyl glycolate** |  |  | **FSA L107A/L163A** | **30** | **-** |  |
| **47** | **Acetic acid** |  |  | **FSA L107A/L163A** | **45** | **-** |  |
| **48** | **Acetaldehyde** | ***D*-glyceraldehyde 3-phosphate** |  | **FSA D6H** | **83** | **(S,S)**  **de:˃98** | [**Roldán et al., 2017**](#_ENREF_45) |
| **49** | **Acetone** |  |  | **FSA D6H** | **85** | **(S,S)**  **de:˃98** |  |
| **50** | **2-Butanone** |  |  | **FSA D6H** | **85** | **(S,S)**  **de:˃98** |  |
| **51** | **Cyclopentanone** |  |  | **FSA D6H** | **82** | **-** |  |
| **52** | **Propionaldehyde** |  |  | **DERA_Arthro_** | **88** | **-** | [**Chambre et al., 2019**](#_ENREF_9) |
| **53** | **Acetone** |  |  | **DERA_Arthro_** | **72** | **-** |  |
| **54** | **Cyclobutanone** |  |  | **DERA_Arthro_** | **74** | **-** |  |
| **55** | **Cyclopentanone** |  |  | **DERA_Arthro_** | **68** | **-** |  |
| **56** | **Glycoaldehyde** |  |  | **DERA_Arthro_** | **89** | **-** |  |
| **57** | **1,3-Dihydro-**  **xyacetone** |  |  | **DERA_Arthro_** | **65** | **-** |  |
| **58** | **Dihydroxyacetone**  **phosphate** | **1,3-Dihydroxyacetone** |  | **RhuA** | **100** | **-** | [**Laurent et al., 2018**](#_ENREF_37) |
| **59** |  | **Hydroxyacetone** |  | **RhuA** | **95** | **-** |  |
| **60** |  | **1-Hydroxy-2-butanone** |  | **RhuA** | **85** | **-** |  |
| **61** |  | **2-Hydroxycyclohexanone** |  | **RhuA** | **84** | **-** |  |
| **62** |  | ***D*-Erythrulose** |  | **RhuA** | **100** | **-** |  |
| **63** |  | **3-Hydroxy-2-oxopropanoic acid** |  | **RhuA** | **92** | **-** |  |
| **64** |  | **Pyruvic acid** |  | **RhuA** | **75** | **-** |  |
| **65** |  | **2,3-Butanedione** |  | **RhuA** | **100** | **-** |  |
| **66** |  | **Acetylacetone** |  | **RhuA** | **83** | **-** |  |
| **67** |  | **Acetoin** |  | **RhuA** | **96** | **-** |  |
| **68** | **3-Fluoropyruvate** | ***L*-Glyceraldehyde** |  | **EcGarL** | **96** | **%(syn)=48** | [**Fang et al., 2019**](#_ENREF_21) |
| **69** |  | ***D*-Glyceraldehyde** |  | **EcGarL** | **97** | **%(syn)=6** |  |
| **70** |  | **(2*S*)-Hydroxypropanal** |  | **EcGarL** | **97** | **%(syn)=90** |  |
| **71** |  | **(2*R*)-Hydroxypropanal** |  | **EcGarL** | **94** | **%(syn)=7** |  |
| **72** |  | **Glycoaldehyde** |  | **EcHpcH** | **100** | **%(syn)=60** |  |
